# Ethnobotanical study of wild edible plants in Dibatie district, Metekel zone, Benishangul Gumuz Regional State, western Ethiopia

**DOI:** 10.1186/s13002-024-00671-2

**Published:** 2024-02-27

**Authors:** Baressa Anbessa, Ermias Lulekal, Paulos Getachew, Ariaya Hymete

**Affiliations:** 1 Department of Plant Biology and Biodiversity Management, College of Natural and Computational Sciences, Addis Ababa University, PO Box 3434, Addis Ababa, Ethiopia; 2 Department of Biology, College of Natural and Computational Sciences, Bule Hora University, Bule Hora, Ethiopia; 3 Center for Food Science and Nutrition, College of Natural and Computational Sciences, Addis Ababa University, Addis Ababa, Ethiopia; 4 Department of Pharmaceutical Chemistry and Pharmacognosy, College of Health Sciences, Addis Ababa University, Addis Ababa, Ethiopia

**Keywords:** Dibatie district, Ethnobotany, Food security, Indigenous knowledge, Wild edible plants

## Abstract

**Background:**

Plants deliver livelihood and food for millions of people in the world. Indeed, wild edible plants support rural communities in developing countries to overcome seasonal unfavorable conditions. In rural areas of Ethiopia, wild edible plants play an indispensable role in fighting food insecurity as emergency or supplementary foods. Hence, this research was aimed at studying the ethnobotanical assessment of wild edible plants in Dibatie district, Metekel zone, western Ethiopia.

**Methods:**

Ethnobotanical data was collected using a semi-structured interview, field observation, focus group discussions, a market survey, and the ranking of selected plants. Besides, voucher specimens were collected and stored at the National Herbarium of Ethiopia. Descriptive statistics, preference ranking, direct matrix ranking, and familiarity index were computed for data analysis.

**Results:**

This study has documented 54 wild edible plant species belonging to 33 plant families and 46 genera. Of these, most (38.90%) had tree growth habits. Wild edible plants bear mostly fruits (72.20%) as edible parts. Local people usually consume these plants freshly raw as complementary foods, though some wild edibles require processing. They were mostly harvested in the January (31.48%) and May (27.78%) months, with the least collected in September (7.41%). Most wild edible plants (78.57%) were available in uncontrolled habitats, while others (21.43%) live in farmlands, home gardens, and as live fences. Out of the recorded plants, about 98% had additional uses besides their nutritional values.

**Conclusion:**

Wild edible plants assist the livelihoods of the local people in food security, agriculture, energy sources, construction, medicines, ecological services, aesthetics, income generation, and household utensils. Nevertheless, wild edible plants are recently threatened due to various anthropogenic factors in the study area. Thus, they need wise use and in-situ and ex-situ conservation measures from all the concerned bodies for sustainable use in the future.

## Introduction

Plants supply livelihood and food in the form of non-timber forest products (NTFPs) for about 300 million people worldwide [1]. These NTFPs are used as food and medicine, especially in tropical and low-income countries [2]. For instance, in areas of poverty and malnutrition, forests serve as the source of edible wild mammals, birds, reptiles, insects, wild spinaches, wild mushrooms, and wild fruits that supplement domestic diets [3]. Predominantly wild edible plants are crucial for human diet, especially in poor rural communities during periods of food scarcity [4].

Wild edible plants contribute a lot to households in rural Africa by decreasing their sensitivity to environmental changes and coping with less favorable conditions [5]. Because they mitigate malnutrition of micro- and macronutrients in addition to their role in enhancing food security [6]. Since some wild edible plants (WEPs) are rich in essential nutrients, they can be used to generate dietetic diversity and avoid overdependence on limited food resources [7, 8]. Besides, they play an important role in income generation for certain local residents [9]. Numerous wild edible plants are believed to have positive effects on human health owing to their medicinal functions [10]. These plants are called ‘nutraceutical plants’ since they provide both nutritional and pharmaceutical benefits [11].

In Ethiopia, WEPs are very helpful to combat food insecurity during periods of hardship (e.g., war, drought, low crop production, etc.) as emergency or supplementary foods [7]. Malnutrition and food insecurity are still among the major humanitarian crises in some parts of the country [12]. Some rural people rely on wild edible plant resources for dietary supplementation and income generation during famine periods [9]. However, the diversity of WEPs is gradually decreasing with the associated indigenous knowledge due to various factors such as agricultural expansion, overgrazing, misuse, and less attention to the preservation of indigenous knowledge [13–15]. Besides, WEPs are underestimated among different indigenous people, as they are considered foods of the poor in some communities of the country [6, 8, 16, 17], and the associated knowledge is transmitted by oral means [9]. They are much more marginalized to document and conserve due to the changes in food habits and lifestyles [18] and the emphasis many researchers placed on cultivated cereal, oil, and industrial crops in the current Ethiopia [19]. As a result, WEPs need safeguarding attention from researchers, governmental and non-governmental administrators, policymakers, local communities, community leaders, religious leaders, and other stakeholders, along with the associated indigenous knowledge.

Although some studies [9, 12, 15, 17, 20–26] have progressed to document WEPs in different regions of Ethiopia, there is still a need for documentation and investigation of new research concerning WEPs due to their nutritional, medicinal, economic, ecological, and other uses, as well as the incidence of threats currently facing them. The study area, Dibatie district in Metekel zone, is rich in ethnic and plants diversity [27]. Different ethnic groups in the area consume WEPs as dietary supplements, famine resilience, and confectionery foods in addition to their other uses. However, the literature indicates that these crucial wild food plants in the present study area are not documented with the associated indigenous knowledge up-to-date. Therefore, this study was aimed at investigating the ethnobotany of wild edible plants in Dibatie district, Metekel zone, Benishangul Gumuz Regional State, western Ethiopia.

## Materials and methods

### Study area

**Fig. 1:**
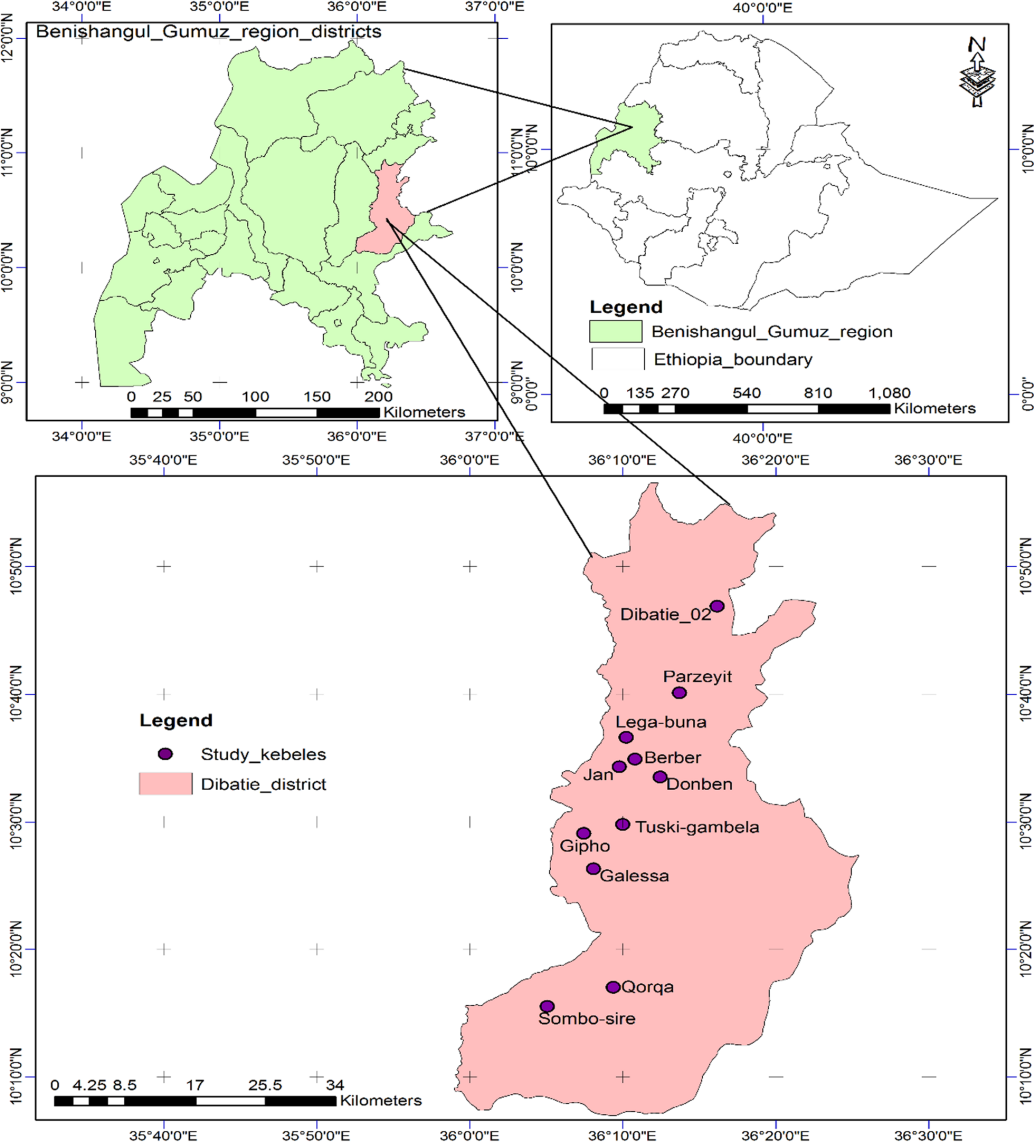
Map of Ethiopia showing Benishangul Gumuz Regional State and the study district (details of the study sites are also presented in Table 1)

The study was conducted in Dibatie district, Metekel zone, Benishangul Gumuz Regional State in western Ethiopia (Fig. 1). Dibatie district is among the seven administrative districts in Metekel zone. It is located about 550 km north-west of Addis Ababa, which is the capital city of the country. The district contains a total of 30 kebeles (the smallest administrative units in Ethiopia), which are 25 rural kebeles and 5 town kebeles. The current study was carried out in 11 kebeles, which are Dibatie_02, Parzeyit, Lega-buna, Berber, Jan, Donben, Gipho, Tuski-gambela, Galessa, Qorqa, and Sombo-sire kebeles (Fig. 1). The district is inhabited by Agaw, Amhara, Gumuz, Oromo, and Shinasha ethnic groups. Out of these, however, Gumuz ethnic groups have not participated in this study due to a long-lasting ethnic conflict that burst in the area during data collection.

**Fig. 2:**
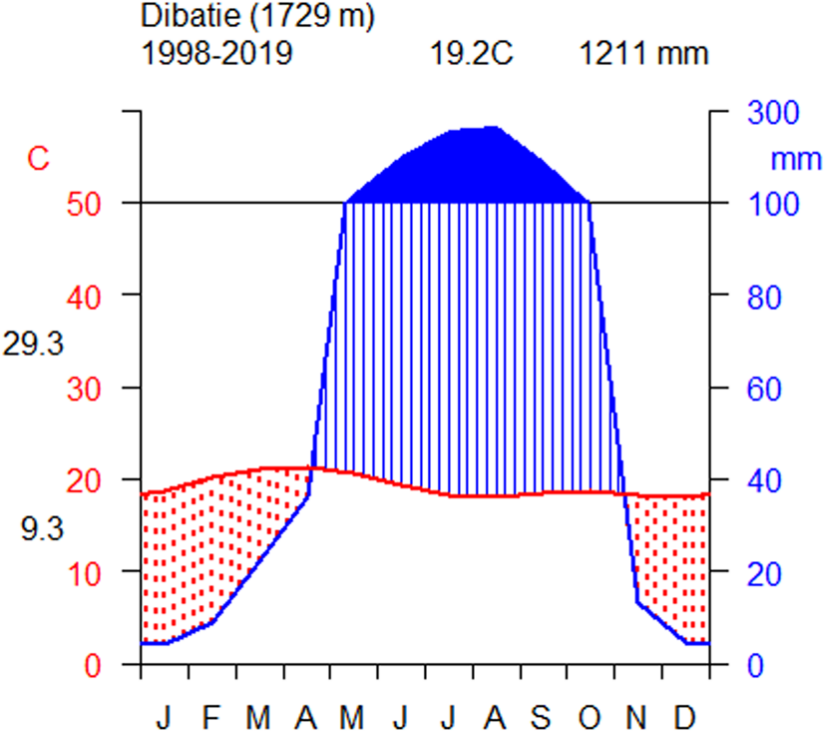
Climadiagram showing mean annual temperature and rainfall from 1998 to 2019. *Data source*: National Meteorological Services Agency, 2022

The study area is found in the elevation range of 1475–2370 m above sea level. It has a mono-modal rainfall pattern, which occurs usually from May to October, with maximum rainfall appearing in July and August. The average annual rainfall in the area is 1211 mm, and the mean annual minimum temperature is 9.3 °C in the highlands, while the mean maximum temperature is found to be 29.3 °C in the lowland areas (Fig. 2). Agro-ecologically, the study area includes lowland (Qola), mid-highland (Weyina-Dega), and highland (Dega) agro-climatic zones.

The livelihood of the local community is mainly dependent on rain-fed cereal-based mixed agricultural systems [28]. Hence, the local people practice traditional lifestyles, including the cultivation of staple food crops (e.g., maize, finger millet, sorghum, tef, lablab, vegetables, cherry pepper, pumpkin, and beans), fruits (e.g., mango, papaya, and banana), oil crops (e.g., groundnut, linseed, niger seed, sesame, castor, and sunflower), and the husbandry of livestock, such as cattle, donkeys, sheep, and goats [27, 29]. Additionally, some (especially Gumuz people) practice ancient activities, i.e., hunter-gatherer and inactive agricultural lifestyles [27].

### Sampling methods and socio-demographic characteristics of informants

The study sites (kebeles) were selected purposefully based on the geographic variations, native ethnic group composition, security of the area, and road access to the study site. Accordingly, 11 kebeles with 9879 total households were selected as representative study sites. Out of these householders, a total of 374 informants were selected as representative respondents using the formula developed by Cochran’s (1977) at a 95% confidence level [30]. The representative informants include 220 randomly selected general householders and 154 purposively selected key informants, such as community elders, administrative leaders, and religious leaders. Accordingly, 34 informants were interviewed from each kebele.

In perspective of their socio-demographic feature, 274 male and 100 female informants participated in the interview. Out of the total of 374 informants, Agaw, Amhara, Oromo, and Shinasha ethnic groups were represented by 10, 64, 158, and 142 respondents, respectively. These ethnic groups were found differently within the study sites (kebeles) and participated in the data collection accordingly. The informants speak Amharic, Agawgna, Afaan oromoo, and Shinashigna languages, in which a particular individual can speak more than one language. Regarding their religion, informants follow Islam (45, 12.03%), Orthodox (163, 43.58%), and Protestant (166, 44.39%) religion types. Informants in the age group greater than 18 years old participated in the interview. Accordingly, respondents of age categories 18–40 years (109, 29.14%), 41–60 years (167, 44.65%), 61–80 years (87, 23.26%), and above 80 years (11, 2.94%) were interviewed. Most (319, 85.29%) informants were farmers, some (28, 7.49%) were civil servants, some others (23, 6.15%) were merchants, and a few (4, 1.07%) were laborers. The details of the socio-demographic profiles of interviewees are presented in Table 1.

**Table 1:** Sampled administrative kebeles with their position, elevation, number of householders, and socio-demographic profiles of interviewed informants

Kebele (sub district)	Latitude	Longitude	Altitude (m asl)	NH	Socio-demographic characteristics of informants								
					General informants		Key informants		Eth (Ag, Am, Or, Sh)	Lan (Ac, Agn, Ao, Shi)	Rel (Is, Ort, Pr)	Age (18–40, 41–60, 61–80, > 80 years)	Occ (Cs, Fa, La, Me)
					M	F	M	F					
Berber	10° 34′ 54″ N	36° 10′ 48″ E	1599	1261	13	7	11	3	Am, Or, Sh	Ac, Ao, Shi	Is, Ort, Pr	10 (18–40), 16 (41–60), 8 (61–80)	Cs, Fa, Me
Dibatie_02	10° 46′ 53″ N	36° 16′ 10″ E	1503	937	14	6	9	5	Ag, Am, Or, Sh	Ac, Agn, Ao, Shi	Is, Ort, Pr	9 (18–40), 18 (41–60), 7 (61–80)	Cs, Fa, La, Me
Donben	10° 33′ 30″ N	36° 12′ 27″ E	1597	1091	14	6	10	4	Ag, Am, Or, Sh	Ac, Agn, Ao, Shi	Is, Ort, Pr	11 (18–40), 15 (41–60), 7 (61–80), 1 (> 80)	Cs, Fa
Galessa	10° 26′ 18″ N	36° 08′ 05″ E	1627	1687	13	7	9	5	Am, Or	Ac, Ao, Shi	Is, Ort, Pr	11 (18–40), 19 (41–60), 4 (61–80)	Cs, Fa, Me
Gipho	10° 29′ 05″ N	36° 07′ 27″ E	1678	864	15	5	11	3	Am, Or, Sh	Ac, Ao, Shi	Ort, Pr	8 (18–40), 19 (41–60), 4 (61–80), 3 (> 80)	Cs, Fa, Me
Jan	10° 34′ 18″ N	36° 09′ 47″ E	1601	485	14	6	11	3	Am, Sh	Ac, Ao, Shi	Is, Ort, Pr	12 (18–40), 12 (41–60), 8(61–80), 2 (> 80)	Fa, Me
Lega-buna	10° 36′ 36″ N	36° 10′ 14″ E	1535	489	16	4	12	2	Am, Sh	Ac, Ao, Shi	Is, Ort, Pr	9 (18–40), 15 (41–60), 9(61–80), 1 (> 80)	Cs, Fa
Parzeyit	10° 40′ 06″ N	36° 13′ 42″ E	1567	809	15	5	12	2	Am, Sh	Ac, Ao, Shi	Is, Or	11 (18–40), 14 (41–60), 7 (61–80), 2 (> 80)	Cs, Fa, Me
Qorqa	10° 17′ 01″ N	36° 09′ 23″ E	1704	1154	11	9	10	4	Or, Sh	Ac, Ao, Shi	Ort, Pr	11 (18–40), 12 (41–60), 11 (61–80)	Cs, Fa, Me
Sombo-sire	10° 15′ 31″ N	36° 05′ 04″ E	2079	483	14	6	11	3	Or, Sh	Ac, Ao, Shi	Ort, Pr	10 (18–40), 13 (41–60), 11 (61–80)	Cs, Fa
Tuski-gambela	10° 29′ 47″ N	36° 10′ 00″ E	1560	619	17	3	12	2	Or, Sh	Ac, Ao, Shi	Ort, Pr	7 (18–40), 14 (41–60), 11 (61–80), 2 (> 80)	Cs, Fa
Total				9879	156	64	118	36					

*NH* Number of householder, *M* Male, *F* Female, *Eth* Ethnicity, *Ag* Agaw, *Am* Amhara, *Or* Oromo, *Sh* Shinasha; Language (Lan), *Ac* Amharic, *Agn* Agawgna, *Ao* Afaan oromoo, *Shi* Shinashigna; Religion (Rel), *Is* Islam, *Ort* Orthodox, *Pr* Protestant; Occupation (Occ), *Cs* Civil servant, *Fa* Farmer, *La* Laborer, *Me* Merchant

### Data collection

The ethnobotanical information was collected by using a semi-structured interview, field observation, market survey, and ranking of selected plants based on their roles to fight famine and multiple uses [31]. In addition, a focus group discussion was conducted in each kebele with the community elders, cattle herders, and school boys, as described in [32].

The interview was carried out in Afaan oromoo, Agawgna, Amharic, and Shinashigna languages, with the help of translators, while the researchers were not able to understand the native languages of participants. The interviews focused on the edible parts, harvesting time, occasions and modes of consumption, adverse effects, and additional uses of wild edible plants. Additionally, it included the local name of plants, marketability, threats to, and management methods of wild edible plants in the study area. The field survey was conducted through guided field observation, focus group discussions, and the collection of voucher specimens for identification. One group discussion, with five to ten participants, was conducted in each kebele with the key informants. The discussion emphasized the traditional uses, preferences, and conservation practices of common WEPs in their locality. Later, the identification of voucher specimens was carried out at the National Herbarium of Addis Ababa University (ETH). The identified botanical names were confirmed by taxonomic experts at ETH and then cross-checked for taxonomic updates by referring to the website Plants of the World Online (POWO).

### Data analysis

The collected ethnobotanical data were analyzed through descriptive statistics (percentage and frequency distribution). Preference ranking and direct matrix ranking were applied to assess the degree of preference of selected wild edible plants [31]. The familiarity index (Fi) was calculated to indicate the relative popularity of wild edible plants within the community [33] using the formula: ${\text{Fi}} = \frac{{\text{Number of times a given species was mentioned as food}}}{{\text{Total number of respondents}}} \times 100$. Microsoft Excel version 2013 and R-statistical packages (climatol, ggplot2, scales, and dplyr) were used for data analysis.

## Results and discussion

### Taxonomic diversity of wild edible plants

The study has documented 54 wild edible plant species belonging to 33 plant families and 46 genera. The family Rubiaceae was represented by 5 plant species (9.26%), followed by the Malvaceae, Moraceae, Myrtaceae, and Solanaceae families, each containing 3 edible plant species (5.56%). All the other families were denoted by two or less wild edible plant species. The genus *Ficus* had relatively the highest number (3 wild edible plant species), followed by *Dioscorea, Grewia, Rumex, Searsia, Solanum,* and *Syzygium*, each of which contains 2 species, while each of the remaining genera was represented by a single species (Table 2).

**Table 2:** List of WEPs including their local names, families, growth habits, edible parts, modes of consumption, additional uses and habitats

Scientific name	Local name (La)	Family	GH	EP	MoC	AU	Ha	Citation in Ethiopia	VN
*Amaranthus caudatus* L	Ambartifo (AO)	Amaranthaceae	H	S	Po	Bf	Fl,Ho	[13, 22, 38]	BA-194
*Ampelocissus schimperiana* (Hochst. ex A. Rich.) Planch	Laalu (AO)	Vitaceae	C	F,Ys	Fr	Fo	Rv	[7]	BA-164
*Annona senegalensis* Pers	Bamburxaa (AO)	Annonaceae	T	F	Fr	Fw,Bf	Ow,Gl	[7, 39]	BA-132
*Borassus aethiopum* Mart	Guchii (AO)	Arecaceae	T	F,Ys	Fr,Ck	Fo,O	Ho,Sa	[25, 36, 40, 41]	BA-47
*Bridelia scleroneura* Müll.Arg	Bacancuwa (AO)	Phyllanthaceae	T	F	Fr	Fw	Ow,Rv	[13, 32, 36]	BA-186
*Canarina abyssinica* Engl.^*^	Xuuxo-rooba (AO)	Campanulaceae	C	N	Fr	M	Rv	[42]	BA-203
*Capparis tomentosa* Lam.^*^	Gimero (Am)	Capparidaceae	Sh	F	Fr	M	Rv	[14, 43]	BA-9
*Carissa spinarum* L.^*^	Agamssa (AO)	Apocynaceae	Sh	F	Fr	Fe,Bf,M,Fw	Ow,Rv,Gl	[9, 12, 15, 17, 20–24, 37, 44, 45]	BA-28
*Corchorus olitorius* L	–	Malvaceae	H	L,Ys	Ck	Bf,Fo	Fl,Sa	[14, 19, 21, 32, 36, 38, 40, 46, 47]	BA-198
*Cordia africana* Lam.^*^	Wanza (Am)	Boraginaceae	T	F	Fr,Dr	Ti,Fo,Fw,Bf,M,U	Fl,Ho	[9, 12, 15, 17, 21–26, 37]	BA-142
*Dioscorea praehensilis* Benth.^*^	Eecaa (AO)	Dioscoreaceae	C	Tu	Bo,R	M	Ow,Rv	[38, 48–50]	BA-3
*Dioscorea schimperiana* Hochst. ex Kunth	Barodaa (AO)	Dioscoreaceae	C	Tu	Bo,R	-	Rv	[7, 22, 51]	BA-75
*Diospyros abyssinica* (Hiern) F. White	Ciinciroo (AO)	Ebenaceae	T	F	Fr	Fw,Fo	Rv,Fm	[14, 21, 34, 47]	BA-103
*Embelia schimperi* Vatke^*^	Hanquu (AO)	Primulaceae	Sh	F	Fr	M,Bm	Rv	[9, 17, 22, 24, 26, 37, 42, 50, 52, 53]	BA-118
*Ensete ventricosum* (Welw.) Cheesman	Baala-warqee (AO)	Musaceae	H	F	Fr	U	Ho,Sa	[15, 26, 36, 54]	BA-79
*Eugenia bukobensis* Engl	Dhala-badda (AO)	Myrtaceae	T	F	Fr	Fe	Rv	[53]	BA-88
*Ficus sur* Forssk.^*^	Fuka (Shi)	Moraceae	T	F	Fr,Dr	Fo,U,M,Sf,Ch,Co	Rv,Gl	[12, 14–17, 20–26, 37, 45]	BA-155
*Ficus sycomorus* L.^*^	Odaa (AO)	Moraceae	T	F	Fr,Dr	Fo,U,M,Sd,Bt,Ch,Sf	Ow,Fl,Gl	[12, 14–17, 20, 22, 24, 25, 37]	BA-33
*Ficus vasta* Forssk	Qilxuu (AO)	Moraceae	T	F	Fr,Dr	Sd,U	Rv,Fm,Sa	[16, 17, 20, 22–26, 36, 37]	BA-144
*Flacourtia indica* (Burm.f.) Merr	Akukku (AO)	Salicaceae	Sh	F	Dr	U	Ow	[9, 17, 22, 39, 41, 55]	BA-173
*Flueggea virosa* (Roxb. ex Willd.) Royle^*^	Baskaya (Shi)	Phyllanthaceae	Sh	F	Fr	M,Fo	Ow,Gl	[13, 21, 25, 34, 40, 47, 54]	BA-13
*Gardenia ternifolia* Schumach. & Thonn.^*^	Gambelloo (AO)	Rubiaceae	T	F	Fr	Fo,Fw,U,M	Ow,Gl	[14, 17, 25, 32, 36, 55]	BA-31
*Grewia ferruginea* Hochst. ex A. Rich	Dhoqunu gurracha (AO)	Malvaceae	Sh	F	Fr	U	Rv,Fm	[9, 12, 15, 17, 21, 24, 25]	BA-102
*Grewia mollis* Juss.^*^	Qoriya (Shi)	Malvaceae	T	B,F	Fr,Ck	Bf,M,U,Lu,Ts	Ow,Rv	[11, 14, 23, 32, 34, 36]	BA-35
*Justicia schimperiana* (Hochst. ex Nees) T. Anderson.^*^	Simiza (Am)	Acanthaceae	Sh	N	Fr	M,Fe,Bf	Lf,Sa	[7, 36, 42]	BA-159
*Keetia zanzibarica* (Klotzsch) Bridson	Shumbura boyye (AO)	Rubiaceae	Sh	F	Fr	Fw	RV,Fm	Not reported	BA-109
*Lantana camara* L	Kusayee (Ao)	Verbenaceae	Sh	F	Fr	Fe,O	Lf,Sa	[12, 34, 43, 54]	BA-162
*Lippia abyssinica* (Otto & A.Dietr.) Cufod	Kusayee durbaa (AO)	Verbenaceae	Sh	F	Fr	Bf	Fm	Not reported	BA-171
*Mimusops kummel* Bruce ex A.DC	Qolaxii (AO)	Sapotaceae	T	F	Fr	Fw,Bf,U,Fo	Rv	[9, 12, 17, 20, 22, 24–26, 37, 55, 56]	BA-89
*Momordica foetida* Schumach.^*^	Qorii-arragessa (AO)	Cucurbitaceae	C	F,L,Ys	Fr,R	M,Fo	Lf,Sa	[14, 22, 36, 37, 42, 53, 55]	BA-72
*Mussaenda arcuata* Poir	Futfutii (AO)	Rubiaceae	C	F	Fr	Fw	Rv	[7, 39, 53]	BA-131
*Nauclea latifolia *Sm.^*^	Kurumba (Shi)	Rubiaceae	T	F	Fr	M	Ow,Gl	[25]	BA-119
*Oxytenanthera abyssinica* (A.Rich.) Munro	Shimel (Am)	Poaceae	Cu	Ys	Ck	Co,Fe,Fo,U,Bm	Ho,Rv	[7, 36, 57]	BA-154
*Phoenix reclinata* Jacq.^*^	Mexxi (AO)	Arecaceae	T	F,Th	Fr,Ck	U,Fo,M,Tb	Rv	[14, 15, 17, 20, 22, 24, 25]	BA-26
*Physalis lagascae* Roem. & Schult	Awut (Am)	Solanaceae	H	F	Fr	Bf	Fl,Ho	Not reported	BA-16
*Piliostigma thonningii* (Schumach.) Milne-Redh	Lillu (AO)	Fabaceae	T	F	Dr	Bf,Fo,Fw	Ow,Fl,Gl	[14, 22, 32, 36, 41, 54, 58]	BA-32
*Psychotria orophila* E.M.A.Petit	Bururii (AO)	Rubiaceae	Sh	F	Fr	Fe	Rv,Fm	Not reported	BA-86
*Rubus apetalus* Poir	Goraa (AO)	Rosaceae	Sh	F	Fr	Fe	Rv,Fm	[15, 17, 22, 26, 39, 42]	BA-97
*Rumex abyssinicus* Jacq.^*^	Dhangaggo (AO)	Polygonaceae	H	Tu	Sp	M	Sa	[12, 20, 22–24, 26, 37, 53]	BA-135
*Rumex nervosus* Vahl^*^	Embacho (Am)	Polygonaceae	Sh	Ys	Fr	M	Ho,Sa	[12, 15, 17, 22–24, 26, 59]	BA-24
*Saba comorensis* (Bojer ex A.DC.) Pichon	Wenno (AO)	Apocynaceae	C	F	Fr	Bm,U,Co,Bf	Rv	[14, 21, 25, 36, 39, 40]	BA-17
*Searsia glutinosa* (Hochst. ex A.Rich.) Moffett	Xaaxessaa (AO)	Anacardiaceae	Sh	F	Fr,Dr	Fe,Fw	Rv	[15, 17, 23, 26, 34, 47]	BA-188
*Searsia ruspolii* (Engl.) Moffett	Xaaxessaa (AO)	Anacardiaceae	Sh	F	Fr,Dr	Fo,Fw,Fe	Ow,Gl	[32, 36, 39]	BA-48
*Senna petersiana* (Bolle) Lock^*^	Sarar-qamale (AO)	Fabaceae	Sh	F	Fr,Dr	M	Ow,Fm	[53]	BA-80
*Solanum lycopersicum* L	Qumadoro xiqqo (AO)	Solanaceae	H	F	Fr,Ck	Bf	Ho,Sa	[60]	BA-176
*Solanum villosum* Mill.^*^	Huncha (Shi)	Solanaceae	H	F,L,Ys	Fr,R	M	Fl,Ho	[13, 22, 37]	BA-153
*Strychnos innocua* Delile	Amburqaa (AO)	Loganiaceae	T	F	Fr	Fw,Fo	Ow,Rv	[25, 36, 37, 39, 54]	BA-157
*Syzygium guineense* (Wild.) DC. subsp. *guineense*	Baddessaa (AO)	Myrtaceae	T	F	Fr	Fw,Co,Bf,Sd,Cm,Ch	Rv	[13, 17, 32, 36]	BA-66
*Syzygium guineense* (Wild.) DC. subsp. *macrocarpum* (Engl.) F.White	Goosu (AO)	Myrtaceae	T	F	Fr	Ch,Fw,Bf	Ow,Fl,Gl	[17, 36]	BA-7
*Uvaria angolensis* Welw. ex Oliv	Geergisoo (AO)	Annonaceae	Sh	F	Fr	Bm	Rv,Fm	[7]	BA-110
*Vepris nobilis* (Delile) Mziray	Hadheessa (AO)	Rutaceae	T	F	Fr	Fe,Fw	Rv	[17, 42]	BA-106
*Vitex doniana* Sweet	Ququraa (AO)	Lamiaceae	T	F	Fr,Dr	Ch,Sd,Co,Fo,Bf,Bt	Ow,Fl,Gl	[12, 25, 36, 39, 53]	BA-29
*Ximenia americana* L.^*^	Hudhaa (AO)	Olacaceae	Sh	F	Fr	Fw,M,Cm	Ow,Rv	[12, 15, 17, 22–26, 37, 53, 56]	BA-49
*Ziziphus abyssinica* Hochst. ex A. Rich.^*^	Unguga (Shi)	Rhamnaceae	T	F	Dr	M,Fo,U	Ow,Gl	[9, 12, 25, 32, 34, 36, 47]	BA-12

*La* Language, *AO* Afaan Oromoo, *Am* Amharic, *Shi* Shinashigna, *GH* growth habit, *C* Climber, *Cu* Culm, *H* Herb, *Sh* Shrub, *T* Tree, *EP* Edible parts, *B* Bark, *F* Fruit, *N* Nectar, *S* Seed, *Th* Trunk heart, *Tu* Tuber, *L* leaf, *Ys* Young *shoot*, *MoC* mode of consumption, *Bo* boiled, *Ck* cooked, *Dr* dry raw, *Fr* fresh raw, *Po* porridge, *R* roasted, *Sp* spice, *AU* Additional uses, *Bf* Bee forrage, *Bt* Beehive tree, *Bm* beehive materials, *Ch* charcoal, *Cm* cleaning materials, *Co* construction, *Fe* fence, *Fo* fodder, *Fw* fuel wood, *Lu* lubricant, *M* medicine, *O* ornamental, *Sd* shade, *Sf* soil fertilizer, *Ti* Timber, *Ts* Traditional soap, *Tb* tooth brush, *U* utensils, *Ha* habitats, *Fl* farmland, *Fm* forest margin, *Gl* grazing land, *Ho* home garden, *Lf* live fences, *Ow* open woodland, *Rv* Riparian vegetation, *Sa* Settlement areas; Nutraceutical plants (^*^); Voucher number (VN); BA-XY/XYZ [2–3 digital number; BA, Baressa Anbessa; XY, two digital number; XYZ, three digital number]

In this study, the family Rubiaceae was represented by the highest number of wild edible plant species, unlike other studies conducted in Ethiopia [9, 13, 24, 25]. Moreover, the number of WEPs recorded in the study area was higher compared to the number of wild edible plant species documented by other studies [9, 13, 14, 24] in various parts of Ethiopia. The higher number of WEPs in the study area indicates the diversity of wild edible plant resources and the dependence of the local people on their consumption [17]. The relatively higher diversity of WEPs in the specific area also implies a strong interaction between the plants and the local communities [34]. In addition, the number of preserved WEPs depends on the proximity of the area to the city and the lifestyles of the residents [35].

Compared to the previous ethnobotanical studies in Ethiopia, the studies conducted in Midakegn district, central Ethiopia [17], Soro district, southern Ethiopia [22], and Bullen district, northwest Ethiopia [36] reported the most overlaps of WEPs (18 species each) recorded in the present study. In addition, the study conducted by [25] in lowland areas of Ethiopia reported high (16 species) overlaps of WEPs with the current study, followed by [12] in North Wollo, northeastern Ethiopia (12 species), [24] in Sedie Muja district, northwest Ethiopia (12 species), [37] in Ensaro district, central Ethiopia (12 species), [14] in Quara district, northwest Ethiopia (11 species), [15] in Berek district, central Ethiopia (11 species), and [26] in Yilmana Densa and Quarit districts, northwest Ethiopia (11 species). Whereas all the other ethnobotanical studies showed less than 11 overlapping wild edible plant species (Table 2). The similarity in the distribution of WEPs along with their traditional use might be due to the geographical similarity and the cultural sharing between local communities inhabiting different areas [9]. However, some WEPs could be used differently in various parts of the country. For instance, the seed powder of *Amaranthus caudatus* L. was eaten being prepared into porridge in the present study and in Burji district, southern Ethiopia [13], and reported to be consumed as a leafy vegetable in Soro district [22] and in Derashe and Kucha districts of southern Ethiopia [38].

Out of the 54 recorded WEPs in the study area, 4 species, such as *Keetia zanzibarica* (Klotzsch) Bridson, *Lippia abyssinica* (Otto & A.Dietr.) Cufod., *Physalis lagascae* Roem. & Schult., and *Psychotria orophila* E.M.A.Petit were not reported in the previous studies conducted in different parts of Ethiopia, as well as in 413 WEPs reviewed by [7]. On the other hand, some WEPs, such as *Carissa spinarum* L., *Cordia africana* Lam., *Ficus sur* Forssk., and *Ximenia americana* L., were highly reported by the previous studies and widely consumed in many areas of the country.

### Growth forms of wild edible plants

**Fig. 3:**
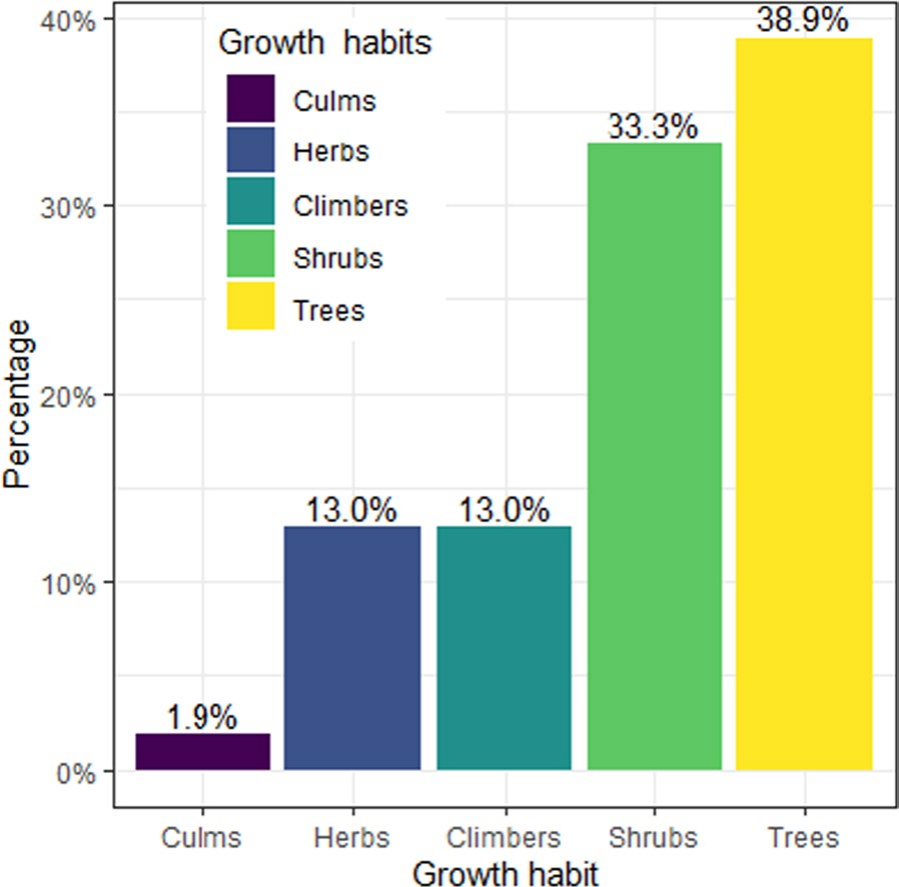
Growth habits of wild edible plants in the Dibatie district

Wild edible plants in the study area exhibit different growth forms. Out of the reported WEPs, most plant species were trees (38.90%), followed by shrubs (33.30%), climbers (13.00%), herbs (13.00%), and culms (1.90%) (Fig. 3). In the current study, wild edible trees were dominant, in line with the studies conducted by [15] in Berek district, central Ethiopia, [21] in Metema district, northwestern Ethiopia, and [22] in Soro district, southern Ethiopia. However, studies conducted in Mieso district, eastern Ethiopia [9], Burji district, southern Ethiopia [13] and Sedie Muja district, northwestern Ethiopia [24] reported the dominance of shrubs, and the study by [26] in Yilmana Densa and Quarit districts, northwest Ethiopia, stated the dominance of wild edible herbs. The similarity or variation in growth habits of WEPs might be owing to their distribution in similar or different climatic conditions, topographies, and altitudes [9, 24].

### Edible parts of wild edible plants in the area

**Fig. 4:**
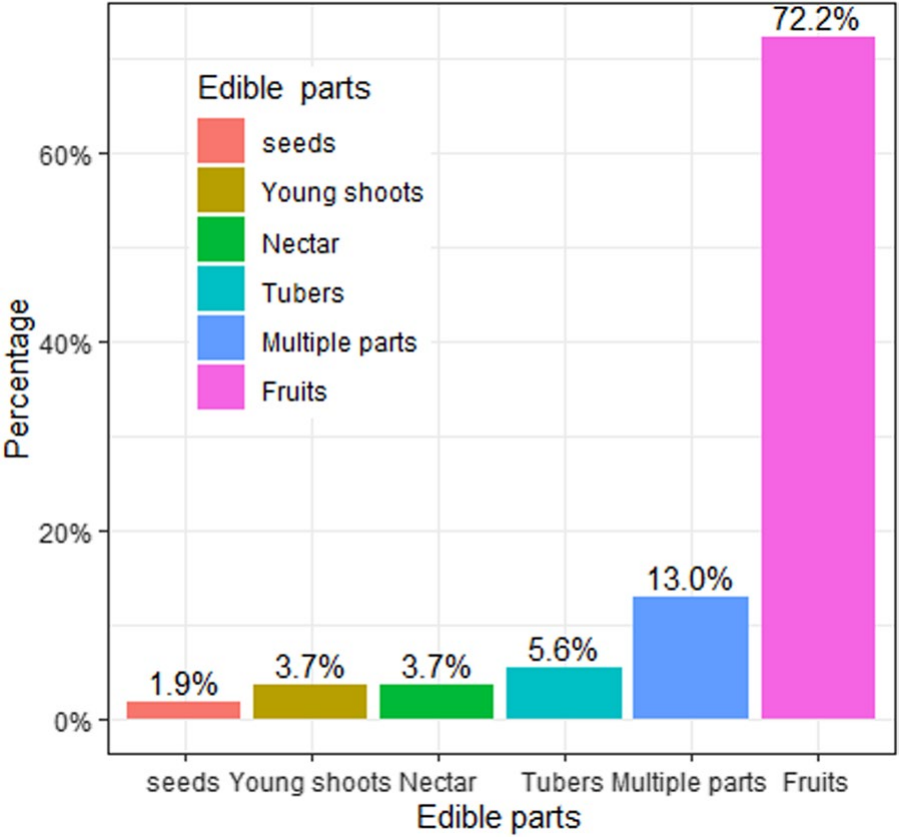
Edible parts of wild edible plants in the Dibatie district

Wild edible plants in the study area have various edible parts, mostly fruits (72.20%), followed by multiple edible parts (13.00%), tubers (5.60%), nectar (3.70%), young shoots (3.70%), and seeds (1.90%) (Fig. 4). Multiple edible parts are those in which two or more parts of a single species are eaten. For instance, the bark and fruits of *Grewia mollis* Juss., the trunk heart and fruits of *Phoenix reclinata* Jacq., and the leaves, young shoots, and fruits of *Solanum villosum* Mill. were consumed by different ethnic groups at the same or different times in the area (Table 2).

Fruits were the predominantly eaten wild edible plant parts (72.20%) in the study area, similar to the dominance of fruit edibility mentioned in the reports of other studies [9, 13, 14, 20] across the country. This might be due to the ease of consuming fruits raw, without processing, by livestock herders, travelers, and schoolchildren. Moreover, the consumption of multiple edible parts of a single plant (13.00%) was reported in the current study, consistent with the previous findings reported by [7]. Such plants have a potent effect on food security in that they provide alternative edible parts at different periods of the year for society [21]. In the study area, for example, young leaves and shoots of *Momordica foetida* Schumach. were eaten roasted, usually by Gumuz ethnic groups and sometimes by Oromo and Shinasha communities, and its ripe fruits were eaten uniformly by all ethnic groups in the area. Previous studies [14, 22, 37, 42, 55] conducted in other parts of the country also confirmed the consumption of *M. foetida* fruits in different communities. However, its boiled tuber is consumed as a root vegetable by the Meinit community living in Bench-Maji zone, southwest Ethiopia [53]. This indicates the potency of *M. foetida,* which needs to be promoted and developed as a leafy and root vegetable as well as a fruit-bearing plant at the national level throughout the country. On the other hand, the current study and other studies [26, 36, 54] conducted in northwest Ethiopia reported *Ensete ventricosum* (Welw.) Cheesman as a wild edible plant in which its ripe fruits were edible rather than its stem. Besides, other studies conducted in north-central [15] and west-central Ethiopia have reported *E. ventricosum* as an underutilized wild edible plant with edible fruit and stem. In the southern parts of Ethiopia, however, it is cultivated and taken as a staple or co-staple food crop, and its processed stem is regularly consumed by about 25 million people throughout the year [61]. It is also reported that *E. ventricosum* is rich in dietary starch and fiber content and less susceptible to short-term climate changes [62]. Therefore, the habits of its management and utilization practices in the southern parts of Ethiopia needs to be expanded to other parts of the country in order to enhance its role in food security.

In the present study, the bark mucilage of *G. mollis* was prepared into the edible souse with spices such as *Allium sativum, Capsicum annuum*, *Foeniculum vulgare*, *Zingiber officinale,* and salt and eaten with porridge or local bread (Enjera), mainly by Gumuz and Shinasha ethnic groups and rarely by others. Small-scale farmers consume the souse of its bark mucilage as a substitute for homemade stew during normal times and during short-term food scarcity. The bark mucilage consumption of *G. mollis* was previously reported only by the studies conducted in Bullen district, northwest Ethiopia [36] and in the Amaro Special District of southern Ethiopia [34], while the fruit edibility was highly described by other studies [11, 14, 23, 32] in the country. In other African countries, a ripe fruit is edible in Uganda [63], a bark mucilage is used as an additives in local cake preparation, and a bark powder is used to improve the texture of foods in Nigeria [64]. This shows the vital attributes of *G. mollis* in traditional food preparations and fighting against food insecurity in Ethiopia as well as in Africa.

### Harvesting periods of wild edible plants

**Fig. 5:**
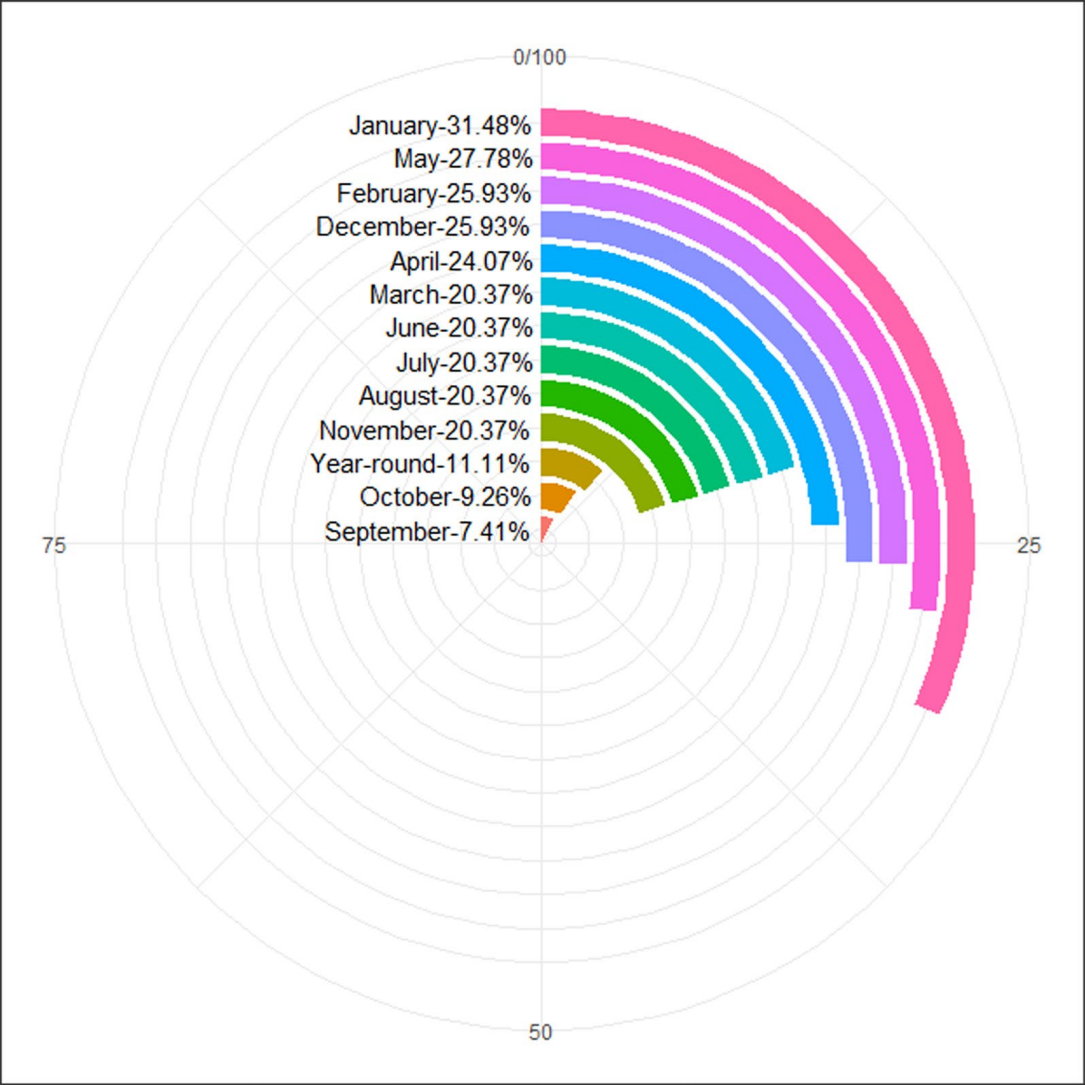
Harvesting periods of wild edible plants in the Dibatie district

Harvesting periods indicate the seasonal availability of wild edible plants in the area. The majority of WEPs in the area were harvested in January (31.48%), May (27.78%), February (25.93%), December (25.93%), April (24.07%), March (20.37%), June (20.37%), July (20.37%), August (20.37%), and November (20.37%), and rarely collected in October (9.26%) and September (7.41%). Certain wild edible plants (11.11%) are still eaten throughout the year (Fig. 5). For example, tubers of *Dioscorea* spp. (*D. praehensilis* Benth. and *D. schimperiana* Hochst. ex Kunth), the trunk heart of *P. reclinata*, the bark of *G. mollis,* and the young shoots of *Borassus aethiopum* Mart. were consumed year-round.

This finding revealed that WEPs were mainly collected in the January, May, February, and December months and relatively rare in September and October. Contrary to the current study, the study conducted by [23] in Raya-Azebo district, northern Ethiopia, described September and October as the main harvesting months for wild edible plants. In another study [9], March, April, and May months were reported as the major harvesting periods in Mieso district, eastern Ethiopia. February and March were also reported by [16] as the major collecting months for wild edible plants. Thus, the seasonal availability of WEPs might vary slightly from region to region in Ethiopia, and this could be due to variations in the agro-ecological and climatic conditions [12, 25, 35, 38] and dissimilarity in plant species [54]. Herbaceous edible vegetables are usually available during the rainy season and sometimes in irrigated areas during the dry season [38]. Whereas shrubby and tree edible fruit species are harvested mainly during the dry season [39].

Similar to the present finding, the study conducted by [9] has described some WEPs that were collected throughout the year. These year-round harvested plants may have a huge contribution to reduce food insecurity due to their availability during all seasons of the year [14, 65]. Indeed, the above-described examples of year-round WEPs in the present study area were reported to be consumed mainly during seasonal food shortages and to relieve hunger during normal times. Therefore, they have the ability to combat famine and food insecurity in the current study area and throughout the country.

### Mode of consumption

The local communities in the study area are endowed with indigenous knowledge of how to use wild edible plants. Usually, they consumed wild edible fruits and vegetables freshly raw without any processing (31 species, 57.41%). Local people sometimes ate wild edible plants in a fresh, raw, or dry state (8 species, 14.81%). Besides, some wild plants were eaten fresh raw or cooked (4 species, 7.41%), dry raw (3 species, 5.56%), boiled or roasted (2 species, 3.70%), cooked (2 species, 3.70%), fresh raw or roasted (2 species, 3.70%), and in porridge (1 species, 1.85%) and spice (1 species, 1.85%) forms (Table 3).

**Table 3:** Mode of consuming wild edible plants in the area

Mode of consumption	No. of wild edible plants	Percent (%)
Fresh raw	31	57.41
Fresh/dry raw	8	14.81
Fresh raw/cooked	4	7.41
Dry raw	3	5.56
Boiled/roasted	2	3.70
Cooked	2	3.70
Fresh raw/roasted	2	3.70
Porridge	1	1.85
Spice	1	1.85

Wild edible plants were usually consumed freshly or dry as emergency or supplementary foods by livestock herders, travelers, schoolchildren, and fuel wood collectors. Likewise, outdoor consumption of unprocessed WEPs was reported in different parts of the country in agricultural fields, during cattle herding, traveling, and firewood collection [13, 17, 32]. However, some plants require different preparation techniques to be consumed at home or at work. Particularly green leafy vegetables and tubers need to be boiled, roasted, or cooked before consumption. Because cooking was required to reduce the level of anti-nutritional factors, such as phytates, tannins, and oxalates in green vegetables and tubers [66]. Besides, seeds of *Amaranthus caudatus* were reported to be powdered with cultivated cereals (e.g., *Eleusine coracana* (L.) Gaertn., *Eragrostis tef* (Zucc.), etc.) and eaten as porridge during the shortage of staple food. This result is consistent with the study conducted by [13] in Burji district, southern Ethiopia, indicating the similarity in the indigenous knowledge of the local people living about thousand kilometers away from each other. On the other hand, tubers of *Rumex abyssinicus* Jacq. were processed as spice with other plants like *Allium sativum*, *Coriandrum sativum*, *Foeniculum vulgare,* and *Zingiber officinale* even during normal periods. Informants confirmed that it gives a yellowish color to the spice used in food stew. Similarly, the study conducted by [37] in Ensaro district, north-central Ethiopia, reported the use of powdered tuber as a spice in cooking food. The other studies conducted in North Wollo [12] and Raya-Azebo district [23], northern Ethiopia, reported the use of *R. abyssinicus* tuber as tea spice, and other studies conducted in Yilmana Densa and Quarit districts, northwest Ethiopia [26], and in Chelia district, west-central Ethiopia [42], revealed the use of its tuber for butter refining and as an ingredient for local tea preparation. In addition, several researchers [20, 22, 24, 42, 53] verified that the young shoots of *R. abyssinicus* are consumed raw in different regions of the country. This implies the diverse nutritional roles of WEPs and the rich associated indigenous knowledge of the local communities living in different areas.

### Occasions of consuming wild edible plants

**Fig. 6:**
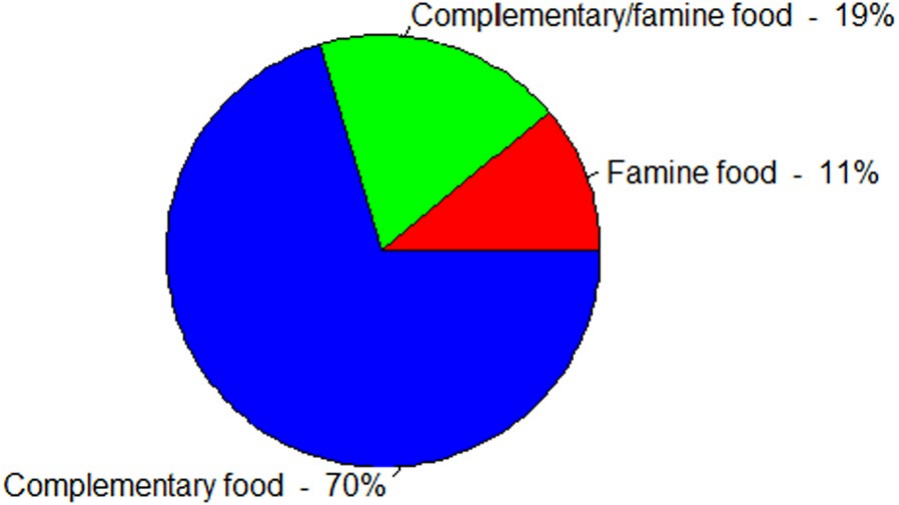
Consuming occasions of wild edible plants in the Dibatie district

Results showed that WEPs in the study area were eaten as complementary foods (70%), both as complementary and famine foods (19%), and as famine foods (11%) (Fig. 6). Here, local communities consume the majority (38 species, 70%) of WEPs mainly as additional foods due to their pleasant tastes, though staple foods are plentiful. However, some WEPs (16 species, 30%) in the study area were consumed to relieve hunger while spending time at schools, farming places, and livestock herding, and during the scarcity of staple food staff in the area. Thus, local communities depend on WEPs as alternative foods and as famine foods to fill the food gaps during the scarcity of popular crops. In agreement with the present study, the study conducted by [14] reported the consumption of nearly 70% of WEPs as supplementary foods and a few (about 35%) WEPs as famine or drought foods. However, contrary to the current finding, other studies [9, 40] conducted in different areas of Ethiopia have reported that most WEPs are consumed as famine foods. This implies that consumption of WEPs might be more dependent on the productivity and/or access to popular crops in different parts of the country [54, 60]. Moreover, the collection and consumption of WEPs depend on the wealth status of households [58]. This indicates that the lower the wealth category of the households, the more they are dependent on WEPs, and vice versa. Such habits of the local people underestimate the nutritional values of WEPs and their contribution to food security.

As the study area consists of three types of agro-ecological zones (lowland, mid-highland, and highland areas) with sufficient monomodal rainfall, the frequency of drought-induced famine is lower compared to other lowland and semi-arid areas of the country, like the Afar, Somali, and southern Oromia regions. As a result, most (70%) WEPs in the area are reported to be consumed as supplementary foods. On the other hand, informants verified that some WEPs, such as *Amaranthus caudatus*, *Corchorus olitorius* L., *Dioscorea schimperiana*, and *Oxytenanthera abyssinica* (A.Rich.) Munro, were eaten only during the seasonal food shortage but not during the normal periods. Especially *A. caudatus* and *C. olitorius* were considered weeds of cultivated crops during normal times. In other parts of the country, however, these plants are commonly eaten and prepared in the form of various products. For example, in southern Ethiopia, the seed powder of *A. caudatus* is reported to be consumed in the forms of pan cake, porridge, and local beverages [13], and its leaves and seeds are also eaten cooked [22]. In the Afar region of eastern Ethiopia, young shoots and leaves of *C. olitorius* are consumed cooked as vegetables, and its dried leaves were reported to be sold in supermarkets and exported to the neighboring country (Djibouti) [19]. It is also reported as a preferable leafy vegetable in the southern [38, 40] and the northwestern [14, 21, 47] parts of Ethiopia. This indicates the food source potential of *A. caudatus* and *C. olitorius*, which needs attention from the agriculture and food policies of the country. Because these plants are short-season annual plants, they can be harvested more than once per year.

In the present study, boiled or roasted tubers of *Dioscorea praehensilis* and *Dioscorea schimperiana* were stated to be consumed during seasonal food gaps. *D. praehensilis* was also reported as a favorite supplementary food for rural communities. These tuberous WEPs consist of stored food reserves (carbohydrates) that help with their growth and are rich in essential nutrients [49]. For example, *D. praehensilis* is described as a cheap source of macro- and micronutrients, combating malnutrition in some rural communities in Ethiopia [48, 66]. Moreover, root and tuber crops like sweet potato (*Ipomoea batatas* L.) and *Dioscorea* spp. are drought-resistant and productive even on marginalized lands with low soil nutrients compared to cereal crops, such as maize, wheat, triticale, and so on [67, 68]. Thus, it is better to domesticate and propagate such multiple stress-resistant and nutrient-full tuberous WEPs to reduce hunger, malnourishment, and poverty, and to use them consistently in the future. That is because it is essential for developing countries like Ethiopia to look for alternative food sources in order to ensure sustainable food and nutrition security [19].

A preference ranking was carried out by seven key informants to rank seven commonly used WEPs based on their contribution to combating famine, their taste, and their safety to eat. Key informants were requested to give values (1–7) according to their point of view on the selected plants, where number 7 represents the most likely preferred plant and number 1 denotes the least likely plant. Accordingly, *Syzygium guineense* (Wild.) DC. subsp. *macrocarpum* (Engl.) F.White was ranked 1st, followed by *Syzygium guineense* (Wild.) DC. subsp. *guineense*, *Ximenia americana*, *Vitex doniana* Sweet, *Carissa spinarum*, *Dioscorea praehensilis*, and *Ficus sycomorus* L., with their corresponding ranks of 2nd, 3rd, 4th, 5th, 6th, and 7th, respectively (Table 4). The scores depend on the views of key informants who participated, since the habit of using WEPs varies from person to person.

**Table 4:** Preference ranking of the seven selected wild edible plants according to the views of respondents

Wild edible plants	Respondents (R_1_–R_7_)							Total score	Ranking
	R_1_	R_2_	R_3_	R_4_	R_5_	R_6_	R_7_		
C. spinarum	4	5	4	6	4	4	6	33	5th
*D. praehensilis*	2	3	3	3	1	2	2	16	6th
*F. sycomorus*	1	2	2	2	2	3	1	13	7th
*S. guineense* subsp. *guineense*	6	7	6	5	7	5	6	42	2nd
*S. guineense* subsp. *macrocarpum*	7	6	7	7	6	7	7	47	1st
*V. doniana*	6	4	5	4	5	6	4	34	4th
*X. americana*	5	5	6	5	6	4	5	36	3rd

### Additional uses of wild edible plants in the study area

Out of the reported wild edible plants, about 98% of them had additional uses beyond their food values across the community (Table 2). These additional uses include utilization as bee forages, beehive hanging trees, beehive materials, charcoal, cleaning materials, construction materials, fences, soil fertilizers, fodders, fuel woods, lubricants, traditional medicines, as ornamentals, tooth brushes, shade, timber, traditional soaps, and household utensils. For example, both *S. guineense* subsp. *guineense* and *S. guineense* subsp. *macrocarpum* were important sources of bee forage and charcoal. In addition, *Cordia africana* was reported as an important source of timber, *Phoenix reclinata* was identified as a good raw material for the preparation of traditional mats, and *Oxytenanthera abyssinica* was reported as a nice raw material for the construction of traditional houses and the preparation of household utensils, and figs of *Ficus* spp. (*F. sycomorus*, *F. sur,* and* F. vasta* Forssk.) were used as fodder for livestock and soil fertilizer, among others. Different studies [9, 12–14, 25] in various parts of Ethiopia have also reported additional uses of WEPs besides their food values.

### Direct matrix ranking

Results of direct matrix ranking revealed that out of the selected seven multipurpose wild edible plants, *C. africana, S. guineense* subsp. *guineense,* and *O. abyssinica* were ranked 1st, 2nd, and 3rd, respectively, based on ten use categories. Whereas *F. sycomorus, F. sur, G. mollis*, and *P. reclinata* were ranked 4th, 5th, 6th, and 7th, respectively (Table 5). The use categories were bee forage, charcoal, construction, fence, fodder, food, fuel wood, household utensils, medicine, and shade. Like the current result, other studies undertaken by [13] in Burji district, southern Ethiopia, and [15] in Oromia special zone, central Ethiopia, have ranked *C. africana* as the most commonly used plant in the societies. This indicates the extreme requirements of *C. africana* for several values within the country. However, widely used plants are usually threatened due to overexploitation for various purposes. Studies [13, 23, 25] revealed that WEPs are usually exploited for purposes other than nutritional value. According to the present study, for example, *C. africana* was usually utilized for its timber production and fodder, whereas *S. guineense* subsp. *guineense* and *O. abyssinica* were highly required for construction purposes within the community. As a result, *C. africana* and *O. abyssinica* were highly declined from the natural forests and started to be propagated in the gardens.

**Table 5:** Direct matrix ranking of 7 multipurpose wild edible plants based on 10 use criteria (7, most likely used; 1, least likely used; 0, not used)

Use categories	Wild edible plants							Total score	Rank
	*Cordia africana*	*Ficus sur*	*Ficus sycomorus*	*Grewia mollis*	*Oxytenanthera abyssinica*	*Phoenix reclinata*	*S. guineense* subsp. *guineense*		
Bee forage	7	1	1	6	0	4	7	26	8th
Charcoal	3	5	5	2	1	0	7	23	9th
Construction	6	4	4	3	7	6	7	37	3rd
Fence	2	2	3	2	7	1	3	20	10th
Fodder	7	5	5	4	6	5	1	33	5th
Food	7	6	6	7	6	5	7	44	1st
Fuel wood	5	5	6	5	6	1	7	35	4th
Household utensil	7	6	6	6	7	7	2	41	2nd
Medicinal	7	5	3	5	3	7	2	32	6th
Shade	7	3	7	1	4	3	5	30	7th
Total score	58	42	46	41	47	39	48		
Rank	1st	5th	4th	6th	3rd	7th	2nd		

On the other hand, out of the listed use categories, utilization of WEPs for food, household utensils, and construction were ranked 1st, 2nd, and 3rd, respectively, while the rest of the use categories were, respectively, fuel wood (4th), fodder (5th), medicine (6th), shade (7th), bee forage (8th), charcoal (9th), and fence (10th) (Table 5). Thus, extensive use of WEPs as household utensils, construction materials, fuel wood, fodder, medicine, charcoal, and fence might be considered the major threatening factors of multipurpose plants.

### Familiarity index (Fi) of wild edible plants in the study area

A familiarity index (Fi) was conducted to indicate the relative popularity of the major and minor wild edible plants. Indeed, the top ten WEPs were revealed based on their popularity as wild foods. Accordingly, *S. guineense* subsp. *macrocarpum* was the most popular (Fi = 31.36) wild edible plant, followed by *V. doniana* (Fi = 25.91) in the area (Table 6). These commonly edible wild plants play an important role in combating food insecurity, in addition to the cultivated plants in the area. Especially *S. guineense* subsp. *macrocarpum* provides a high yield and is consumed well for the entire 2 months (April and May) in the study area. Hence, local people usually talk about how children may grow by eating this wild fruit, though staple foods are scarce.

**Table 6:** Familiarity index (Fi) of top ten popular wild edible plants

Wild edible plants	Number of times a species was mentioned	Fi
*Syzygium guineense* subsp. *macrocarpum*	69	31.36
*Vitex doniana*	57	25.91
*Carissa spinarum*	54	24.55
*Cordia africana*	48	21.82
*Ximenia americana*	48	21.82
*Syzygium guineense* subsp. *guineense*	42	19.09
*Saba comorensis*	30	13.64
*Dioscorea praehensilis*	28	12.73
*Ficus sycomorus*	27	12.27
*Ficus sur*	24	10.91

### Nutraceutical wild edible plants

The current finding showed that 21 plant species (38.89%) were reported to have both medicinal and nutritional roles (Table 2). These plants are known as nutraceuticals because their various parts provide dietary and therapeutic values. Out of the reported nutraceuticals, for example, unripe, roasted fruits of *C. spinarum* and fruits of *C.* a*fricana* with seeds were swallowed to expel ascaris, trunk hearts of *P. reclinata* were consumed to relieve malaria, and tubers of *D. praehensilis* were eaten to treat impotency in men. Because nutraceutical plants have phytocomplexes, which play important roles in therapeutic properties owing to the natural active principles they contain [69]. Indeed, WEPs are rich in micronutrients and bioactive secondary metabolites; some also have antioxidant and antimicrobial properties [70]. For instance, *Amaranthus hybridus* L.*, Haplocarpha rueppellii* (Sch.Bip.) Beauverd*, Haplocarpha schimperi* Beauverd*,* and *Rumex nervosus* Vahl [59], as well as *Cleome gynandra* L. and *Solanum nigrum* L. [71], are potential WEPs with good sources of phenolics, flavonoids, and antioxidant agents, treating oxidative stress-related diseases.

In addition to the above examples, informants in the present study confirmed that the decoction of *Embelia schimperi* fruit with seed was commonly used traditionally to treat tapeworm, beyond the nutritional role of a ripe fleshy fruit berry. Similarly, the previous studies [26, 37, 42, 52, 53] reported the nutritional and medicinal contributions of this plant from different areas of the country. This indicates the nutraceutical potential of *E. schimperi* that can be improved in the development of various drugs and food products.

### Marketability of wild edible plants

Results showed that some WEPs were reported to serve as sources of income. Out of the reported WEPs in the area, about 13% were marketable as food or spice in the local market, towns, and schools. For instance, tubers of *R. abyssinicus* were prepared as spice with other spice plants and sold in the local markets. Besides, *X. americana, S. comorensis, G. mollis, V. doniana, B. aethiopum,* and *Mimusops kummel* Bruce ex A.DC. were other marketable WEPs mainly vended in towns and schools for consumption. Wild edible plants were mostly collected and sold by youths, schoolchildren, and sometimes mothers. This is in agreement with the reports of previous studies conducted in different parts of Ethiopia [32, 38, 43]. This implies that community members living in different parts of the country have similar perceptions about consuming and selling wild edible plants.

However, 87% of WEPs were not marketable as foods, although some of them were sold within the community for other purposes or products such as timber, charcoal, mats, construction materials, and so on. Similarly, an earlier study conducted by [26] reported that the majority of WEPs were not marketable owing to their limited access and low habits of consumption compared to domesticated plants. Furthermore, other studies [34, 38] have described the marketability of some WEPs for other products or uses (e.g., timber, agricultural tools, construction, and firewood) rather than nutritional purposes. This indicates an insignificant level of market value chain in the trade of WEPs, though they are highly valuable foods within or outside the country. On the other hand, studies conducted in Quara district [14] and Metema district [21], northwest Ethiopia, reported the export of WEPs, such as *Adansonia digitata* L.*, Balanites aegyptiaca* (L.) Delile*,* and *Tamarindus indica* L., to the neighboring country (Sudan). The report by [19] also revealed the high marketability of dried and packed leaves of *C. olitorius* in the Afar region of eastern Ethiopia and sent them to Djibouti.

## Adverse side effects of consuming wild edible plants

Results showed that the majority (about 78%) of WEPs in the study area did not have adverse effects. Certainly, few plants (22%) were reported to exhibit slightly negative effects on human beings and domestic animals either during or after consumption. The adverse effects were mainly associated with the consumption of unripe fruits, eating with seeds, and excessive consumption. For instance, *Ficus* species were described as causing abdominal pain when excessively consumed with seeds. Besides, unripe fruits of *C. spinarum* and *Rubus apetalus* Poir. resulted in temporary mouth burning, and excessive consumption of *S. guineense* subsp. *macrocarpum*, *C. africana*, *R. apetalus,* and *Senna petersiana* (Bolle) Lock fruits was reported to cause stomach discomforts. Similarly, adverse effects were reported by [54] when WEPs were eaten unripe, in excess, and with an empty stomach. In the current finding, the seeds of *M. kummel* were lethal when consumed by goats. Likewise, earlier studies by [38, 54] have reported that the seeds of *Lepisanthes senegalensis* (Poir) Leenh. were lethal to goats and camels. Therefore, few WEPs require proper handling mechanisms to avoid or reduce the risks of adverse side effects.

Some WEPs in the study area were not easily collected by consumers. For example, *C. spinarum*, *P. reclinata*, *Flacourtia indica* (Burm.f.) Merr., and *Ziziphus abyssinica* Hochst. ex A. Rich. had dangerous thorns and needed to be collected carefully. Besides, some wild edibles like *F. sycomorus,* S. *guineense* subsp. *guineense*, *S. comorensis, V. doniana*,* B. aethiopum*, and *F. vasta* were not easily reached while collecting edible fruits. Similar to the study described by [38], digging edible tubers of *Dioscorea* spp. was a laborious task. Thus, local people use long sticks to collect the fruits of tall trees or climbers and use tough digging materials for underground tubers. This indicates the strong relationship between people and wild plants in the study area.

### Habitats of wild edible plants

The current study showed that the majority (28.57%) of the WEPs were found in riparian vegetation, followed by open woodlands (18.37%). The rest of the WEPs inhabit grazing land (12.24%), settlement areas (10.20%), forest margins (9.18%), farmland (9.18%), home gardens (9.18%), and as live fences (3.06%) (Table 7). A particular wild edible plant can inhabit one or more types of habitats. In agreement with the present finding, other studies [13, 15] have reported various habitats of WEPs, such as riverine areas, woodlands, farmlands, natural forests, grazing lands, live fences, and home gardens. The current finding also described settlement areas as habitats for WEPs, in line with the previous study reported by [9]. Similar to the other studies [9, 12, 13, 20, 24] conducted in Ethiopia, most WEPs were collected from natural habitats and exposed to habitat destruction. However, the availability of some WEPs in farmlands, live fences, home gardens, and settlement areas indicates the promising attempts of local communities toward their conservation.

**Table 7:** Habitats of wild edible plants in the study area

Habitat type	Number of species	Percentage (%)
Riverine	28	28.57
Open woodland	18	18.37
Grazing land	12	12.24
Settlement area	10	10.20
Forest margin	9	9.18
Farmland	9	9.18
Home garden	9	9.18
Live fence	3	3.06

### Threats to and conservation status of wild edible plants

**Fig. 7:**
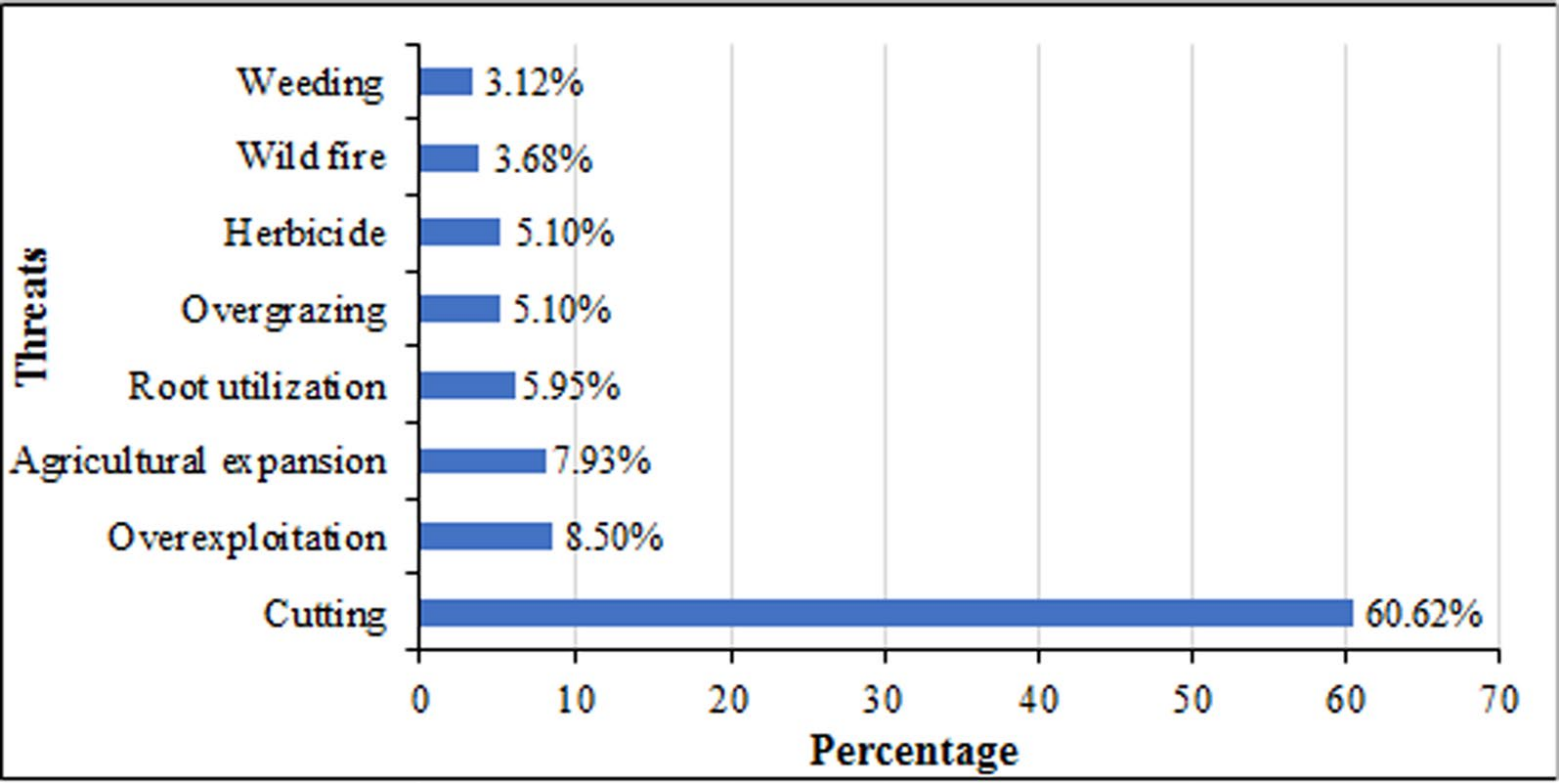
Threats to wild edible plants in the Dibatie district

Informants described that the major threats to WEPs in the study area were cutting (60.62%), overexploitation (8.50%), agricultural expansion (7.93%), root utilization (5.95%), herbicide (5.10%), overgrazing (5.10%), wild fire (3.68%), and weeding (3.12%) (Fig. 7). Local communities cut or deforest WEPs for various purposes, such as food, medicine, fencing, timber, fuel wood, charcoal, construction, fodder, household utensils, and farm materials, among others. Thus, widely used multipurpose WEPs were highly threatened due to their extensive uses for different purposes. This shows that human activities pose the main threats to WEPs in the study area compared to natural disasters. Hence, these plants require wise use and special attention from all the concerned bodies in order to sustain them with associated indigenous knowledge for the next generation. In agreement with the present finding, other studies [9, 12, 13, 15, 26] conducted in Ethiopia have revealed human activities as the major threats to WEPs. On the other hand, the study conducted by [54] in Adiarkay, Debark, and Dejen districts of the Amhara region of northern Ethiopia has reported catastrophic factors as the major threats to wild edible plants.

This finding showed that local communities did not conserve about 76% of the WEPs in the area. Likewise, the previous study conducted by [12] reported that a large number (58.5%) of WEPs were not conserved in Wollo, northern Ethiopia. In the present study area, however, some WEPs (approximately 24%) were slightly conserved due to their extra uses apart from nutrition. For instance, some people keep *C. africana* in gardens or farmland for its timber; a few people cultivate *O. abyssinica* for house construction, fences, preparation of household utensils, and sources of income; and some others conserve *F. sycomorus* for its shade and its use as a beehive hanging tree. In line with the current study, elsewhere in Ethiopia, researchers [9, 13, 15, 25] have reported that some important WEPs were deliberately retained in farmland or propagated in gardens by the local communities. According to the current results, local people believed that some plants, including wild edibles, were contributing to water conservation. For example, *S. guineense* subsp. *guineense* and *F. sur* were considered water-preserving plants since they inhabit riverine or wet environments. Thus, community elders and administrative leaders were inhibiting the destruction of such plants near rivers. Similarly, the study conducted by [15] in Berek district, Oromia special zone, central Ethiopia, stated the roles of social norms and beliefs of the local communities in conserving WEPs onsite. Although the progress is promising, the conservation practices are still very stunted elsewhere in the country [12, 14, 38, 40]. As a result, WEPs are declining from the natural forests from time to time [23].

## Conclusions

The present finding has documented 54 wild edible plant species consumed by the local community living in Dibatie district, western Ethiopia. The majority (70%) of the recorded wild edibles were eaten as alternative foods in the presence of staple foods. However, few (11%) WEPs were consumed only during the scarcity of staple food crops in the area. Thus, the intensity of consuming WEPs depends on access to the cultivated crops. Beside their nutritional supplements, WEPs support livelihoods through beekeeping, energy sources, cleaning materials, construction raw materials, fences, soil fertilizers, livestock fodders, traditional medicines, aesthetics, shade, furniture, farm materials, and household utensils. Moreover, wild Aves and some wild animals were highly dependent on the consumption of wild edible plant resources in the area. Nevertheless, the perception of local people was low toward the conservation, wise use, and management of wild edible plants. Because WEPs are recently threatened by anthropogenic factors (like deforestation, overexploitation, agricultural expansion, root utilization, herbicides, weeding, overgrazing, and human-induced wild fire), they have drastically declined over the last 30–40 years. Therefore, this study calls for an urgent in-situ and ex-situ conservation strategy in order to conserve and transfer wild edible plant resources and associated indigenous knowledge of the local communities to the next generation.

In general, local people in the study area have a strong relationship with WEPs since they use them for nutrition, income generation, traditional medicine, construction, and the preparation of household materials. Hence, wild edible plants require wise use and special conservation attention from all the stakeholders (e.g., local communities, governmental and non-governmental bodies) for sustainable use in the future. Moreover, the national green legacy should include the plantation of indigenous wild edible plants in all regions of the country. Furthermore, the recorded wild edible plants should be verified by conducting further investigations on their nutritional analysis, phytochemical composition, antioxidant activity, and toxicity examination.

## Data Availability

The datasets used and/or analyzed during the current study are available from the corresponding author on reasonable request.

## Abbreviations

ETH: National Herbarium of Addis Ababa University
Fi: Familiarity index
NTFPs: Non-timber forest products
POWO: Plants of the World Online
WEPs: Wild edible plants
